# Genotyping and antibiotic resistance of thermophilic *Campylobacter* isolated from chicken and pig meat in Vietnam

**DOI:** 10.1186/s13099-016-0100-x

**Published:** 2016-05-11

**Authors:** Tuan Ngoc Minh Nguyen, Helmut Hotzel, Hosny El-Adawy, Hanh Thi Tran, Minh Thi Hong Le, Herbert Tomaso, Heinrich Neubauer, Hafez Mohamed Hafez

**Affiliations:** HungVuong University, Viet Tri, PhuTho Vietnam; Institute of Bacterial Infections and Zoonoses, Friedrich-Loeffler-Institut, Jena, Germany; Institute of Tropical Diseases and Zoonoses Vietnam, Hanoi, Vietnam; Institute of Poultry Diseases, Free University Berlin, Berlin, Germany; Institute of Marine Biochemistry, Vietnam Academy of Science and Technology, Hanoi, Vietnam; Department of Poultry Diseases, Faculty of Veterinary Medicine, Kafrelsheikh University, Kafr El-Sheikh, Egypt

**Keywords:** *Campylobacter*, Meat, MLST, Microarray, Antibiotic resistance

## Abstract

**Background:**

*Campylobacter* species are recognized as the most common cause of foodborne bacterial gastroenteritis in humans. In this study nine *Campylobacter* strains isolated from chicken meat and pork in Hanoi, Vietnam, were characterized using molecular methods and tested for antibiotic resistance.

**Results:**

The nine isolates (eight *C. jejuni* and one *C. coli*) were identified by multiplex PCR, and tested for the presence or absence of 29 gene loci associated with virulence, lipooligosaccharide (LOS) biosynthesis and further functions. *fla*A typing, multilocus sequence typing and microarray assay investigation showed a high degree of genetic diversity among these isolates. In all isolates motility genes (*fla*A, *fla*B, *flh*A, *fli*M), colonization associated genes (*cad*F, *doc*B), toxin production genes (*cdt*A, *cdt*B, *sec*D, *sec*F), and the LOS biosynthesis gene *pgl*B were detected. Eight gene loci (*fli*Y, *vir*B11, Cje1278, Cj1434c, Cj1138, Cj1438c, Cj1440c, Cj1136) could not be detected by PCR. A differing presence of the gene loci *cia*B (22.2 %), Cje1280 (77.8 %), *doc*C (66.7 %), and *cgt*B (55.6 %) was found. *iam*A, *cdt*C, and the type 6 secretion system were present in all *C. jejuni* isolates but not in *C. coli*. *fla*A typing resulted in five different genotypes within *C. jejuni*, MLST classified the isolates into seven sequence types (ST-5155, ST-6736, ST-2837, ST-4395, ST-5799, ST-4099 and ST-860). The microarray assay analysis showed a high genetic diversity within Vietnamese *Campylobacter* isolates which resulted in eight different types for *C. jejuni*. Antibiotic susceptibility profiles showed that all isolates were sensitive to gentamicin and most isolates (88.8 %) were sensitive to chloramphenicol, erythromycin and streptomycin. Resistance rates to nalidixic acid, tetracycline and ciprofloxacin were 88.9, 77.8 and 66.7 %, respectively.

**Conclusions:**

To the best of our knowledge, this study is the first report that shows high genetic diversity and remarkable antibiotic resistance of *Campylobacter* strains isolated from meat in Vietnam which can be considered of high public health significance. These preliminary data show that large scale screenings are justified to assess the relevance of *Campylobacter* infections on human health in Vietnam.

## Background

Thermophilic campylobacters are the most common bacterial cause of diarrhoea in humans worldwide [1]. Enteric diseases caused by the thermophilic species *C. jejuni*, *C. coli*, *C. lari*, and *C. upsaliensis* range from asymptomatic infections to severe inflammatory bloody diarrhoea [2]. *C. jejuni* is often associated with the Guillain–Barré syndrome [3]. Virulence mechanisms in campylobacteriosis are currently poorly understood.

Poultry and poultry products remain the most common source of foodborne human campylobacteriosis [4, 5]. The natural habitat of thermophilic *Campylobacter* is the intestinal tract of healthy birds and raw meat that can be contaminated during the slaughtering process. Consumption of undercooked chicken meat or contaminated ready-to-eat food is the most common source of infection. *Campylobacter* are also found in pigs and cattle. Swine carcasses are often contaminated with faeces at the slaughter and processing facilities during the evisceration process, which ultimately leads to contaminated food products [6–8]. Compared to poultry, the relevance of swine in foodborne campylobacteriosis is not well studied. However, a high incidence of *Campylobacter* on pork products at the retail level was found [9, 10].

South East Asia including Vietnam was often considered a hotspot for emerging infectious diseases [11]. Vietnam is currently a developing country and knowledge about *Campylobacter* and campylobacteriosis is limited. Only few data exist about the prevalence of *Campylobacter* in children [12, 13] and adults [14]. The prevalence rates of *Campylobacter* in cases of diarrhea were between 2 and 4 % for children and <1 % for adults. In a study concerning the incidence of diarrhea in rural Vietnamese children [15] *Campylobacter* was the most frequently identified pathogen comprising 31 % of all isolates.

Fifteen to 32 % of meat samples in different regions of Vietnam contained thermophilic *Campylobacter* [16–21]. Duck meat and pork were also contaminated with *Campylobacter* in 23.9 and 53.7 % of tested samples, respectively [21]. Bao et al. isolated thermophilic campylobacters from 35.1 % of chicken carcasses in large and small abattoirs of Ho Chi Minh City and 67.9 % of the isolates belonged to the species *C. jejuni* [22]. However, Schwan investigated meat samples from markets in the Can Tho Province but found no *Campylobacter* spp. [19].

Several molecular biological methods for characterization and discrimination of *Campylobacter* isolates have been developed [23]. PCR and *fla*A typing were used as well as multi-locus sequence typing (MLST) and microarray assays for determination of relatedness among isolates [24, 25].

The molecular genetics of *Campylobacter* has been extensively studied but the pathogenesis of *Campylobacter* infections is not fully understood. A number of putative virulence and toxin genes that may contribute to pathogenicity in human *Campylobacter* infection have partly been identified and sequenced [26–29].

Flagella-mediated motility, adherence to intestinal epithelial cells, invasion and survival in the host cells as well as the ability to produce toxins are important virulence factors [27]. The involvement of the *fla*A gene in *Campylobacter* colonization has been shown [30]. Several *Campylobacter* cytotoxins have been identified [31] and the cytolethal distending toxin (CDT) has been characterized in detail [32, 33]. CDT is composed of three subunits and it has been suggested that CDT, amongst other functions, may play a role in adhesion and invasion [34]. Active CDT is lethal for host enterocytes [35, 36].

It was shown that 19–53 % of *Campylobacter* spp. strains contain plasmids of various sizes [37]. The plasmid-encoded *vir*B11 gene is a marker potentially associated with the virulence of C*ampylobacter* species [38].

A study with Vietnamese isolates dealt with the identification of possible virulence markers like a novel protein translocation system, the type-6 secretion system [39].

The antimicrobial resistance of *Campylobacter* isolates was investigated also in several studies [19, 20, 40]. High resistance rates in *C. jejuni* were determined against ciprofloxacin, nalidixic acid and tetracycline with 64, 46 and 68 %, respectively. Resistance against antibiotics in *C. coli* isolates was higher than in *C.jejuni*. All *C. jejuni* isolates were resistant to ciprofloxacin and nalidixic acid while, 83 % showed resistance to tetracycline [19]. These isolates were recovered from faeces. The broth microdilution method is an easy and reliable method for interpreting minimum inhibitory concentration (MIC) values for *C. jejuni* and *C. coli* which is also recommended by EUCAST [41–43]. The emergence of antimicrobial resistance in *Campylobacter*, particularly to fluoroquinolones, has showed the need for continued monitoring of *Campylobacter* resistance.

In this study, Vietnamese *Campylobacter* isolates were characterized to assess their genetic relatedness, potential virulence factors and antibiotic resistance profiles. The isolates were recovered from chicken and pig meat from two slaughterhouses in Hanoi. The investigation was done using different molecular biological tests, MLST, microarray analysis, and the antimicrobial susceptibility was assessed.

## Methods

### *Campylobacter* isolates

*Campylobacter* were isolated from 100 chicken meat and 50 pork samples of two slaughterhouses in Hanoi, Vietnam, following the International Standards Organization [ISO] 10272-1 (2006) guidelines [44] by the Institute of Veterinary Science in Hanoi, Vietnam, in 2009. Bacteria were stored using the Cryobank system (Mast Diagnostica, Reinfeld, Germany) and transferred to the National Reference Laboratory of Campylobacteriosis at the Institute of Bacterial Infections and Zoonoses of the Friedrich-Loeffler-Institut in Jena, Germany. *Campylobacter* isolates were sub-cultured on Mueller-Hinton Agar (Oxoid GmbH, Wesel, Germany) supplemented with 10 % bovine blood under microaerophilic conditions (5 % O_2_, 10 % CO_2_ and 85 % N_2_) at 42 °C for 48 h. Isolates were kept in cryovials at −80 °C.

### DNA extraction

Genomic DNA was extracted from 48-h bacterial cultures on Mueller-Hinton blood agar plates using the High Pure PCR Template Preparation Kit™ according to the manufacturer’s instructions (Roche Diagnostics GmbH, Mannheim, Germany). Extracted DNA was quantified spectrophotometrically using a Nanodrop^®^ ND-1000 (Fisher Scientific GmbH, Schwerte, Germany). DNA extracts were stored at −20 °C.

### Species confirmation

Bacterial isolates were identified using a multiplex PCR assay [25] targeting the *map*A and *ceu*E genes.

### *fla*A-RFLP typing

*fla*A-RFLP (flagellin A-restriction fragment length polymorphism) typing was performed as described previously [24]. Briefly, a part of the *fla*A gene of the isolates was amplified using primer pair flaA1-Wob/fla2-Wob (Jena Bioscience GmbH, Jena, Germany). The approximately 1700 bp amplicons were digested with *Dde*I (Roche Diagnostics GmbH) as recommended by the manufacturer. The DNA segments were analyzed after electrophoresis on a 1.5 % agarose gel by staining with ethidium bromide and visualization under UV light. Documentation was carried out using a Bio Imaging System (Syngene, Cambridge, UK).

### Multilocus sequence typing

Seven housekeeping gene loci including *asp*A (aspartase A), *gln*A (glutamine synthetase), *glt*A (citrate synthase), *gly*A (serine hydroxyl methyl transferase), *pgm* (phosphor glucomutase), *tkt* (transketolase), and *unc*A (ATP synthase α subunit) were amplified by PCR as described previously [3]. PCR conditions were modified: after initial denaturation at 96 °C for 60 s followed 35 cycles of denaturation at 96 °C for 15 s, annealing at 50 °C for 1 min, and extension at 72 °C for 1 min. Amplicons were examined by gel electrophoresis on a 1.5 % agarose gel and purified with the QIAamp Gel Extraction Kit (Qiagen, Hilden, Germany) according to the recommendations of the manufacturer. Cycle sequencing was carried out using BigDye Terminator v1.1 Cycle Sequencing Kit (Applied Biosystems, Darmstadt, Germany). Analysis of sequencing products was done with a genetic analyzer ABI PRISM 3130 (Applied Biosystems).

Alleles, sequence types (STs), and clonal complexes (CCs) were assigned by submitting DNA sequences of amplicons to the MLST database available at the following website: http://pubmlst.org/campylobacter.

### Microarray DNA hybridization assay

DNA microarray analysis described here was based on the presence or absence of gene loci of *Campylobacter jejuni* isolates using the ArrayTube™ platform (Alere Technologies GmbH, Jena, Germany) [45]. Two types of microarrays with spotted probes were used to differentiate *C. jejuni* isolates: *C. jejuni*-1 and Campy-2. Sample processing was done using a commercial kit (Alere Technologies GmbH) according to the manufacturer’s instructions (www.alere-technologies.com). Briefly, 1 µg of genomic DNA was amplified and labelled by PCR with random primers and biotin-16-dUTP. Labelled DNA was hybridized to both microarrays for 1 h at 45 °C, washed, and quantified after colorimetric reaction using horseradish peroxidase and TrueBlue substrate. Hybridization signals were measured after 5 min precipitation with an ArrayTube transmission reader ATR-03 (Alere Technologies GmbH). Interpretation of array data was described by El-Adawy et al. [24]. SplitsTree analysis was done using BioNumerics (version 4.6; Applied Maths NV, Sint-Martens-Latem, Belgium).

### Molecular biological characterization of *Campylobacter* isolates

Detection of genes which have functions for motility, adhesion, colonization, invasion, toxin production, lipooligosaccharide (LOS) biosynthesis was carried out by PCRs as described previously [29, 46]. The presence of additional gene loci was detected as described in publications cited in Table 1.

**Table 1:** Presence of virulence-associated genes, lipooligosaccharide biosynthesis genes and other gene loci in Vietnamese *Campylobacter* isolates

Species	09CS 0040^a^	09CS 0043^a^	09CS 0046^a^	09CS 0047^a^	09CS 0049^a^	09CS 0067^a^	09CS 0066^b^	09CS 0068^b^	09CS 0051^a^	References
	C.j	C.j	C.j	C.j	C.j	C.j	C.j	C.j	C.c	
Virulence-associated genes										
*fla*A	+	+	+	+	+	+	+	+	+	[29]
*fla*B	+	+	+	+	+	+	+	+	+	[29]
*flh*A	+	+	+	+	+	+	+	+	+	[29]
*fli*M	+	+	+	+	+	+	+	+	+	[29]
*fli*Y	−	−	−	−	−	−	−	−	−	[29]
*cia*B	+	+	+	+	−	+	+	+	−	[29]
*iam*A	+	+	+	+	+	+	+	+	−	[29]
*vir*B11	−	+	−	+	−	−	−	−	−	[29]
*cad*F	+	+	+	+	+	+	+	+	+	[29]
*doc*A	+	+	+	+	+	+	+	+	+	[29]
*doc*B	+	+	+	+	+	+	+	+	+	[29]
*doc*C	−	−	−	−	+	+	+	−	−	[29]
*cdt*A	+	+	+	+	+	+	+	+	+	[29]
*cdt*B	+	+	+	+	+	+	+	+	+	[29]
*cdt*C	+	+	+	+	+	+	+	+	+	[29]
*wla*N	−	−	−	−	−	−	−	−	−	[29]
*cgt*B	−	−	−	+	+	+	+	+	+	[29]
LOS genes										
*pgl*B	+	+	+	+	+	+	+	+	+	[46]
Cje1278	−	−	−	−	−	−	−	−	−	[46]
Cje1280	−	−	−	+	−	+	−	−	−	[46]
Cj1434c	−	−	−	−	−	−	−	−	−	[46]
Cj1138	−	−	−	−	−	−	−	−	−	[46]
Cj1438c	−	−	−	−	−	−	−	−	−	[46]
Cj1440c	−	−	−	−	−	−	−	−	−	[46]
Cj1136	−	−	−	−	−	−	−	−	−	[46]
Secretory genes										
*sec*D	+	+	+	+	+	+	+	+	+	[77]
*sec*F	+	+	+	+	+	+	+	+	+	[77]
Paralogous gene family										
*maf*1	+	+	−	+	+	+	+	+	+	[78]
*maf*4	+	+	+	+	−	+	+	+	+	[78]
Type VI secretion system										
*hcp*	+	+	+	+	+	+	+	+	−	[39]

C.j, *Campylobacter jejuni*; C.c, *Campylobacter coli*

^a^Isolated from chicken meat

^b^Isolated from pork

### Antimicrobial susceptibility testing and MIC determination

The broth micro-dilution test was performed with Sensititre Campylobacter plates EUCAMP 2 (MCS Diagnostics BV, RE Swalmen, The Netherlands). They consist of 96 round-bottom wells which are pre-coated with various concentrations of seven different clinically used antibiotics. The antimicrobial agents and their concentration ranges used in the test are given in Table 2. The susceptibility tests were performed according to CLSI guidelines [41]. Briefly, *Campylobacter* isolates were cultivated on Mueller-Hinton agar (Oxoid GmbH) supplied with 10 % bovine blood under microaerophilic conditions at 37 °C for 48 h. Bacterial colonies were suspended in NaCl solution (0.9 %) for matching turbidity of 0.5 McFarland units (Dr. Lange, CADAS 30 photometer, Berlin, Germany). One-hundred and fifty μl of the suspension were diluted in 10 ml Mueller-Hinton broth (Oxoid GmbH) resulting in a concentration range of 10^6^–10^7^ colony forming units (cfu)/ml. Each well was dispensed with 100 µl of the suspension. The plates were sealed and incubated at 37 °C for 24 h under microaerophilic conditions. The results were obtained by reading either visually or photometrically (Tecan Deutschland GmbH, Crailsheim, Germany) using computer program easyWIN fitting (version V6.1, 2000). *C. jejuni* DSM 4688 (Deutsche Sammlung von Mikroorganismen und Zellkulturen GmbH, Braunschweig, Germany) and *C. coli* DSM 4689 were included in each batch of broth micro-dilution test for quality control.

**Table 2:** MICs and resistance rate of Vietnamese *C. jejuni* isolates

	Concentration range (µg/ml)											R (%)
	<0.06	<0.12	0.25	0.5	1	2	4	8	16	32	64	
Chloramphenicol						6				*2*		25.0
Ciprofloxacin	1	2			*1*	*4*						62.5
Erythromycin				6						*2*		25.0
Gentamicin			3	2	1			*1*	*1*			25.0
Nalidixic acid									1	*1*	*6*	87.5
Streptomycin					1	1	1	*3*	*2*			62.5
Tetracycline			1		1					*6*		75.0

Italic values represent number of resistant isolates

### Molecular biological detection of resistance determinants

#### Erythromycin resistance

Point mutations at positions 2074 and 2075 in domain V of the 23S rRNA were confirmed as the most common mechanism for macrolide resistance in *Campylobacter*. The detection of point mutations was done by MAMA-PCR assay as previously described [47].

#### Ciprofloxacin resistance

A single point mutation (Thr-86-Ile) in the quinolone resistance-determining region (QRDR) of *gyr*A was defined as source of high-level resistance to fluoroquinolones [48]. The MAMA-PCR was done to detect *gyr*A mutation in *C. jejuni* and *C. coli* isolates as described by [49, 50] with modified PCR cycling conditions.

#### Tetracycline resistance

*tet*(O) gene is strongly associated with tetracycline resistance in *C. jejuni*. Primer pair DMT1/DMT2 was chosen to detect this resistance determinant as described previously [51].

PCR conditions were identical with those described by El-Adawy et al. [52].

## Results

### *Campylobacter* species identification

In total, 20 isolates suspected to be *Campylobacter* were cultivated (15 from chicken meat and 5 from pork) in Vietnam, saved cryo-conserved and transferred to Germany. However, only 9 isolates could be re-cultivated on Mueller-Hinton agar. Table 1 gives an overview of origin and species of cultivated *Campylobacter* isolates. Eight isolates belonged to *C. jejuni* and one isolate from chicken meat was identified as *C. coli* by multiplex PCR.

### *fla*A-RFLP typing

**Fig. 1:**
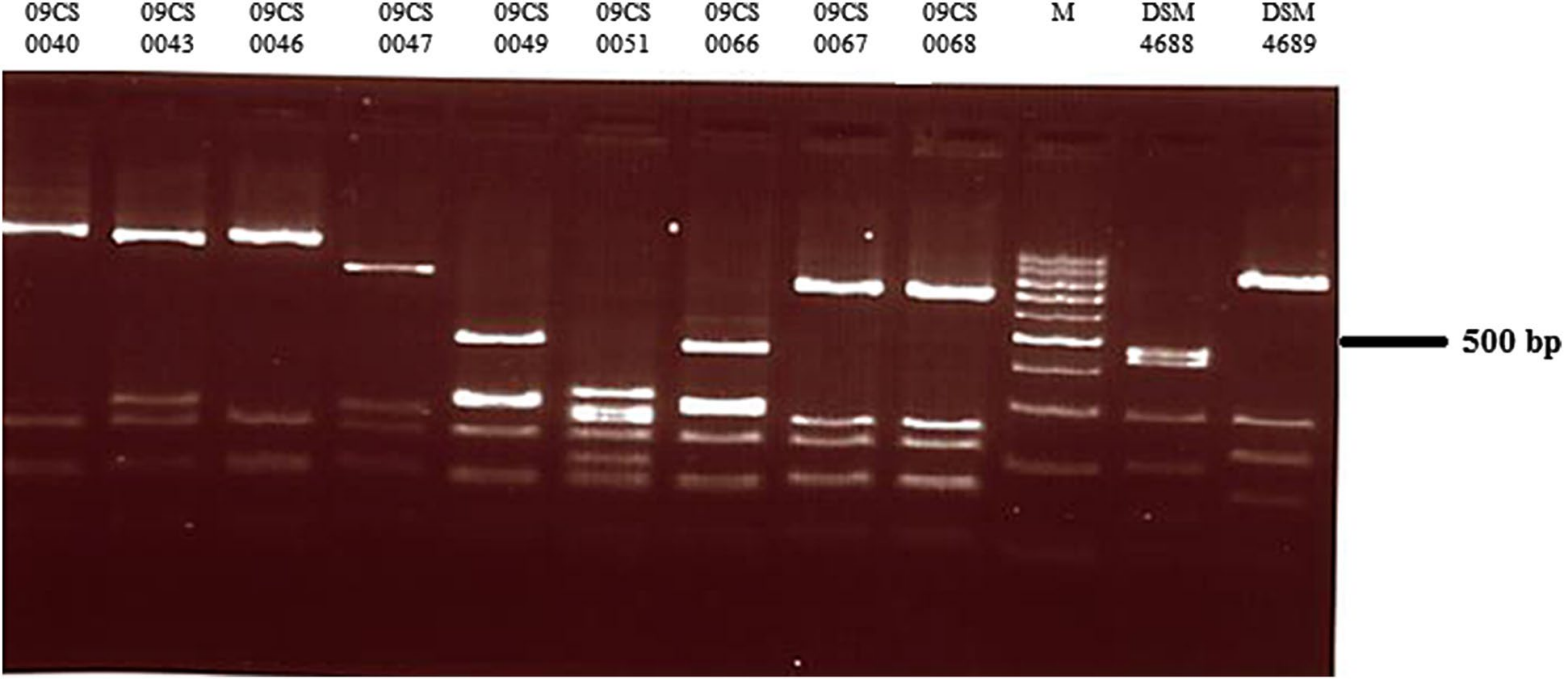
Agarose gel electrophoresis of *fla*A typing products of Vietnamese *Campylobacter* isolates digested with *Dde*I (M—100 bp DNA ladder)

Vietnamese *Campylobacter* isolates were characterized by *fla*A typing using the restriction enzyme *Dde*I (Fig. 1). The restriction profiles of *C. jejuni* yielded five different types.

### MLST

Within the eight *C. jejuni* isolates, six different sequence types were identified (Table 3). Sequence type ST 5799 was found in *C. jejuni* isolates recovered from chicken and pork meat. *C. jejuni* sequence types ST 2837 and ST 4395 found in chicken belonged to the clonal complex ST-353. Four sequence types could be assigned to clonal complexes and two others (ST 5155 and ST 6736) were not assignable.

**Table 3:** Results of MLST of Vietnamese *Campylobacter* isolates

Isolate	*asp*A	*gln*A	*glt*A	*gly*A	*pgm*	*tkt*	*unc*A	ST	CC	Source^a^	Year^a^	Location^a^
09CS0040	1	2	2	212	11	253	147	5155	–	–	–	–
09CS0043	7	2	5	289	10	3	6	2837	ST-353 complex	–	–	–
09CS0046	1	2	2	212	11	253	147	5155	–	–	–	–
09CS0047	24	17	1	2	11	3	6	4395	ST-353 complex	Human	2010	Vietnam
09CS0049	7	2	2	15	23	3	12	5799	ST-443 complex	Human	2010	Japan
09CS0066	7	2	2	15	23	3	12	5799	ST-443 complex	Human	2010	Japan
09CS0067	9	30	2	2	11	59	6	4099	ST-460 complex	Human	2009	Canada
09CS0068	73	2	250	10	10	59	291	6736	–	Human	2013	Thailand
09CS0051	33	39	30	79	113	47	17	860	ST-828 complex	Human, Poultry, Food	1999–2014	USA, Europe, not Asia

^a^Equivalent types from the MLST database

*Campylobacter coli* isolate 09CS0051 belonged to clonal complex ST-828.

### Microarray DNA hybridization assay

**Fig. 2:**
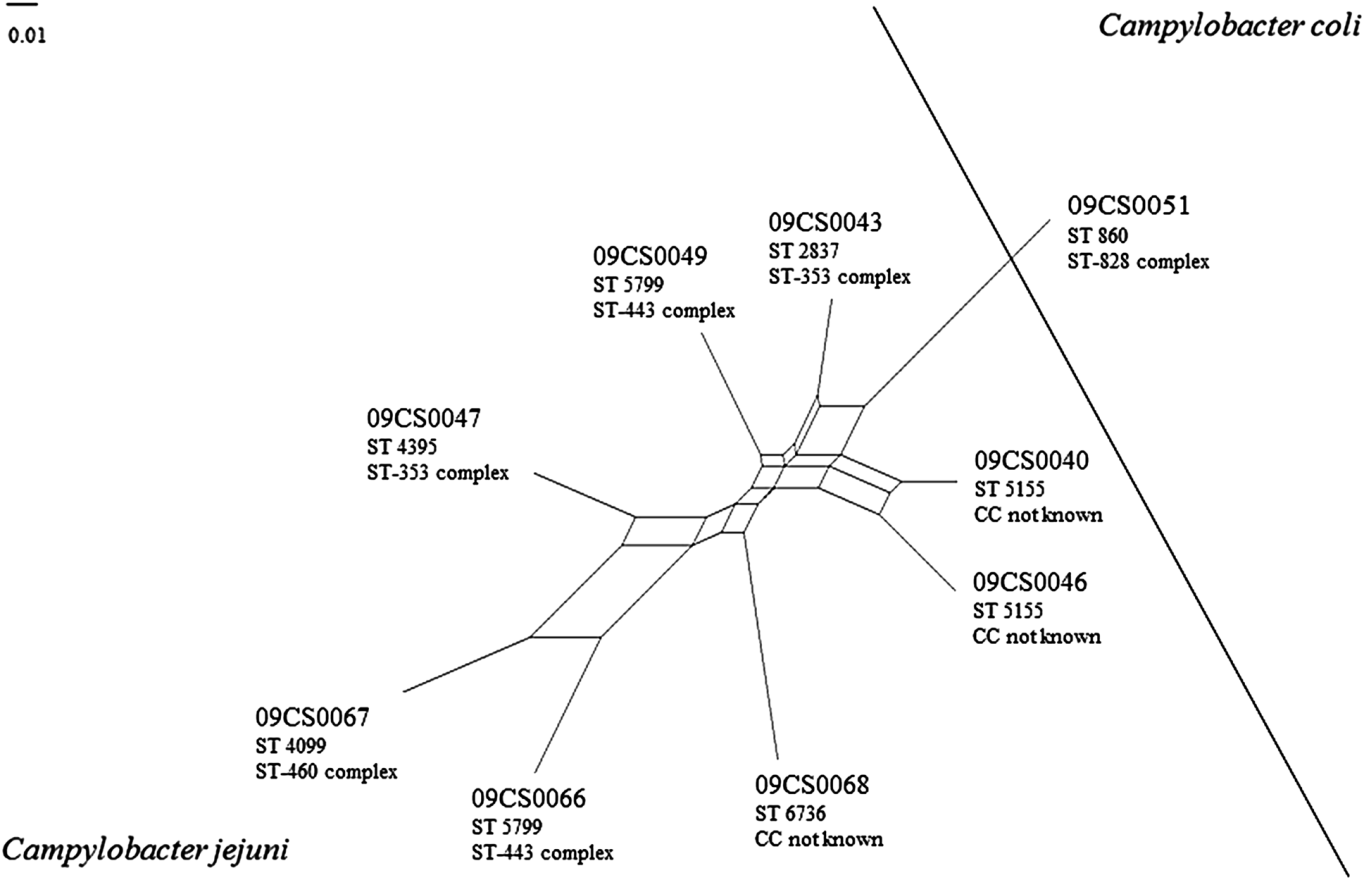
SplitsTree analysis of Vietnamese *Campylobacter* isolates according to microarray investigation

The DNA microarray assay showed high significant genetic diversity among 8 *C. jejuni* isolates (Fig. 2). Isolates 09CS0040 and 09CS0046 were closely related and represented the same sequence type in MLST in which no assignment to an existing clonal complex was possible. Otherwise, isolates 09CS0049 and 09CS0066 belonged to the same sequence type (ST 5299) and CC (ST-443) proved to be different when considerably more gene loci were analyzed using the microarray. Likewise, isolates 09CS0043 and 09CS0047 showed large disparity in microarray analysis independent from their affiliation to ST-353 complex, whereby the different sequence types had to be considered.

### Molecular biological detection of different virulence-associated and toxin genes

*fla*A, *fla*B, *flh*A and *fli*M as genes of the flagellar system of *Campylobacter* were found in all isolates, but *fli*Y could not be detected by PCR (Table 1). The invasion-associated gene *iam*A was present in all *C. jejuni* isolates whereas *cia*B was absent in one *C. jejuni* isolate. The gene *vir*B11 was found in two isolates. All *C. jejuni* isolates carried *cad*F (an outer-membrane protein gene), *cdt*A, *cdt*B and *cdt*C-C (cytolethal distending toxin), *doc*A (encoding a periplasmic cytochrome C peroxidase), and *doc*B (encoding a methyl-accepting chemotaxis protein). Detection of *doc*C (another methyl-accepting chemotaxis protein) was variable among the isolates (Table 1). It was detected in three out of eight isolates. *wla*N (a beta-1,3 galactosyltransferase) which is responsible for a specific LOS structure was not identified in any of the isolates. In contrast, *cgt*B (another beta-1,3 galactosyltransferase gene) was found in five of eight *C. jejuni* isolates. The *C. coli* isolate 09CS0051 showed a difference to the *C. jejuni* strains. The invasion-associated genes *cia*B and *iam*A were not detected by PCR. The presence of LOS biosynthesis genes was determined to characterize the Vietnamese campylobacters. In all *Campylobacter* isolates *pgl*B (encoding a putative oligosaccharyl transferase) was detected. Gene loci for putative galactosyltransferases (Cje1278, Cje1280, Cj1136, Cj1138, Cj1434c, Cj1438c, Cj1440c) were rarely found. Only Cje1280 was detected in two *C. jejuni* isolates.

Secretory genes *sec*D and *sec*F were detected in all *Campylobacter* isolates. Type-6 secretion system (T6SS), a novel class of protein translocation system, was identified over the haemolysin co-regulated protein (*hsp*) gene in all *C. jejuni* isolates.

The motility accessory factor (*maf*) family represents a new class of bacterial genes related to flagellar biosynthesis and phase variation. *maf*1 and *maf*4 were found in almost all isolates with the exceptions of 09CS0047 which lacked *maf*1 and 09CS0049 where *maf*4 could not be identified by PCR.

### Antimicrobial susceptibility testing

The results of antimicrobial susceptibility testing for seven antibiotic agents are given in Table 4. None of the *Campylobacter* isolates was fully susceptible to all investigated antibiotics. The *C. jejuni* isolates were highly resistant to ciprofloxacin, nalidixic acid, streptomycin and tetracycline with 62.5, 87.5, 62.5 and 75.0 % resistance, respectively. The resistance rate for chloramphenicol, erythromycin and streptomycin was low with 25.0 %. *C. coli* isolate 09CS0051 was resistant to ciprofloxacin, nalidixic acid, streptomycin and tetracycline.

**Table 4:** Resistance of Vietnamese *Campylobacter* isolates to various substances

Isolate	Agent						
	CHL	CIP	ERY	GEN	NAL	STR	TET
09CS0040	S	S	S	S	R	S	S
09CS0043	R	R	R	AMB	R	S	R
09CS0046	S	S	S	S	S	AMB	S
09CS0047	S	R	S	S	R	AMB	R
09CS0049	S	R	S	S	R	S	R
09CS0066	S	R	S	S	R	R	R
09CS0067	R	R	R	R	R	R	R
09CS0068	S	S	S	S	R	R	R
09CS0051	S	R	S	S	R	R	R

*CHL* chloramphenicol, *CIP* ciprofloxacin, *ERY* erythromycin, *GEN* gentamicin, *NAL* nalidixic acid, *STR* streptomycin, *TET* tetracycline, *R* resistant, *S* sensitive, *AMB* ambiguous

Molecular biological assays for detection of ciprofloxacin, erythromycin, and tetracycline resistance determinants confirmed the results of phenotypic testing of antimicrobial resistance.

## Discussion

As in other countries, thermophilic *Campylobacter* are common bacterial agents in Vietnam which cause gastro-enteric illness in humans, especially in children [13, 15]. Meat and meat products serve as the main sources of human infections. Huong et al. showed that approximately 30 % of raw chicken samples from school and hospital canteens and retail markets in Hanoi city were contaminated with *Campylobacter* [17]. Other studies from different regions of Vietnam came to comparable results [20, 21]. Here, chicken and pork meat samples from a market in Hanoi were investigated for the presence of thermophilic campylobacters. 13.3 % of the samples were *Campylobacter* positive. *C. jejuni* and *C. coli* were detected as contaminants of chicken and pork meat. In comparison, a report from Germany showed that 52.3 % of chicken carcasses in slaughterhouses and nearly 40 % of raw meat in retail were positive for these microorganisms [53]. It is possible that the lower percentage of *Campylobacter* findings in the meat samples from the Hanoi market is a result of slaughter procedures. In Vietnam the meat is not prepared in large slaughterhouses for retail as in Germany. Chicken and also pigs are slaughtered in low numbers and often directly on-site and the risk of contamination for example by generation of aerosols in slaughterhouses is smaller.

In this study, 20 *Campylobacter* isolates were obtained from meat samples in Vietnam. Unfortunately, after transfer to Germany only 9 isolates could be re-cultivated which were subject to further investigation. Eight of them were identified as *C. jejuni* and one as *C. coli* by mPCR. Several methods were used to type the isolates. As a rapid and simple method to illustrate heterogeneity within the *C. jejuni* isolates, *fla*A-RFLP typing was used. Four different strain types were detected by *Dde*I digestion of the amplified *fla*A gene. This enzyme was used because it showed the highest discriminatory power in former investigations [24] in comparison with *Alu*I or *Sau*3AI. The digestion pattern of *C. coli* isolate 09CS0051 was completely different. Limitations of this typing method resulted from the use of only a very small part of the *Campylobacter* genome and difficulties in standardization of the analytical process. This complicates an inter-laboratory comparison of results between different laboratories.

Microarray analysis worked as a PCR-based comparative genomic fingerprinting (CGF) assay [54] and confirmed the heterogeneity of the *C. jejuni* isolates. An advantage of this method is the use of the whole genome data instead of only one or a few genes. The basis of this method is the detection of the presence or absence of several gene loci that are spread over the whole genome. SplitsTree analysis of the hybridization results showed high genetic diversity as no isolate is identical with another one. The *C. coli* isolate 09CS0051 was clearly distinct from the *C. jejuni* isolates. Additionally, sequence types and clonal complexes of the isolates determined by MLST are given in Fig. 2.

Both methods showed differences concerning the relatedness of different *C. jejuni* isolates among each other. Isolates 09CS0049 and 09CS0066 represented an identical sequence type and belonged to the same clonal complex but in microarray investigation they showed only poor relatedness. 09CS0043 and 09CS0047 were part of the clonal complex ST-353 but differed in the sequence type. Genetic relatedness based on microarray data was marginal. In contrast, two isolates (09CS0040 and 09CS0046) were found with identical sequence type and pattern in *fla*A typing after *Dde*I digestion and even microarray analysis showed a high degree of similarity.

The major advantage of MLST is the comparability of results independent from the laboratory and the local working conditions (technicians, machines etc.). The relatively high costs of this complex technique are outweighed by the hard facts that are obtained in the form of DNA sequences of seven house-keeping genes. In this study, six sequence types in the group of *C. jejuni* isolates were detected. These sequence types were compared with the database on the *Campylobacter* MLST Home Page (http://pubmlst.org/campylobacter/). ST 2837 and ST 4395 belonged to clonal complex ST-353 whereby 09CS0047 (ST 4395) was identical with an isolate which was recovered from a stool sample of a hospital inpatient with gastroenteritis in Vietnam in 2010. 09CS0049 and 09CS0066 belonged to CC ST-443. Sequence type 5799 was previously isolated from human stool samples in Japan. Three isolates could not be assigned to any known clonal complex. The sequence type of isolate 09CS0068 had previously been discovered once in a human stool sample in Thailand, two others were not described yet. Isolate 09CS0067 represented sequence type 4099. This type belongs to the ST-460 complex and was previously identified in a human sample in Canada. *C. coli* isolate 09CS0051 belonged to sequence type 860 and ST-828. Identical isolates were found several times during the last two decades in Europe and the USA. Records from Asia are lacking until now. In summary, the investigated Vietnamese isolates in their majority seemed to represent strains typical for the Asian region. A route of infection of *Campylobacter* from meat to humans can be assumed.

The *Campylobacter* isolates were characterized regarding virulence factors associated with adhesion and invasion of host cells. All isolates harboured flagellin genes *fla*A, *fla*B, *flh*A and *fli*M. Similar observations have been reported previously [27, 55]. Molecular genetic approaches with defined mutants showed that *fla*A is essential for colonization [30]. The complex flagellum of *Campylobacter* species is encoded by two tandem-oriented flagellin genes (*flaA* and *flaB*). While the function of the *fla*A gene seems to be fully elucidated, there are many speculations about the function of the *fla*B gene, which may play a role in antigenic variation or influence the motility in various environmental conditions [56]. *fli*Y, a gene of flagellar motor switch proteins, could not be detected.

The *cia*B gene, coding for a *Campylobacter* invasion antigen, was present in most of the *C. jejuni* isolates. It was absent in 09CS0049 and *C. coli* 09CS0051. Another gene which is important in the invasion process of *Campylobacter* to host cells is *iam*A. It was detected in all *C. jejuni* isolates. Carvalho et al. described the detection of the *iam*A gene in 85 % of invasive *C. jejuni* but in non-invasive isolates it is rare [57]. Also the *cad*F gene was detected in all *C. jejuni* isolates. It encodes for an outer-membrane protein which mediates the binding of the bacteria to fibronectin [58]. Based on the results it can be concluded that these Vietnamese isolates represented invasive *C. jejuni* strains.

Cytolethal distending toxin causes direct DNA damage leading to induction of DNA damage checkpoint pathways [35]. The *cdt* gene cluster consists of 3 genes *cdt*A, *cdt*B and *cdt*C. The *cdt* genes were shown to be conserved among different *Campylobacter* strains [59]. Bang et al. observed that the presence of these genes in isolates from different sources exceeds 90 % [27]. In all Vietnamese *C. jejuni* strains isolated from chicken and pork meat the complete *cdt* gene cluster was observed. Rozynek et al. obtained results for *C. jejuni* strains isolated from children with diarrhea and found that *cdtA*, *cdtB* and *cdtC* were present in 98.4, 97.0 and 98.0 % of all isolates, respectively [60]. However, *cdt*C was not detected in *C. coli* isolate 09CS0051 from chicken meat which was in agreement with a previous study [60]. On the other hand, a similar frequency of *cdt* genes and the *cdt* gene cluster was observed in dog and chicken isolates [55]. In this study all investigated isolates harboured the *cdt*B gene. It is indeed generally accepted that the *cdt*B genes are widespread amongst poultry and cattle as well as in human isolates in Denmark, Japan, Poland, and Belgium [27, 60–62]. However, low percentages of occurrence of *cdt*B have been reported in humans (28 %) and chickens (20 %) in India, which could be due to genetic reasons or variations in the isolates from different geographic areas [63].

Only a minority of *C. jejuni* isolates gave positive PCR results for *vir*B11 encoding a putative component of a type IV secretion system. It is located in the pVir plasmid and could be involved in virulence [38]. The 25.0 % prevalence of the *virB11* gene in *C. jejuni* isolates in this study is higher than 10.3 % in human isolates reported by Bacon et al., but much lower than in pig isolates (35.7 %). Until now, the role of the protein encoded by the *vir*B11 gene in the invasion and colonization process of eukaryotic cells by *Campylobacter* species could not be elucidated.

Macrolides, quinolones and tetracycline are among the common antimicrobials recommended for testing, because they can be of therapeutic relevance in severe cases of infection. High levels of resistance of *Campylobacter* to tetracycline and ciprofloxacin were frequently reported but resistance to erythromycin and gentamicin remained low.

The antimicrobial susceptibility profiles among the Vietnamese isolates were analyzed based on the guidelines of CLSI (2008) [41]. In this study standardization of the protocol for the commercially available broth microdilution test as a method for the determination of the minimum inhibitory concentration (MIC) of antibiotics was done [52]. All isolates were sensitive to gentamicin and most of isolates (88.8 %) were sensitive to chloramphenicol, erythromycin and streptomycin. Similar results were reported in several previous studies [4, 42, 64–68]. In contrast to our findings, a previous study reported high resistance to streptomycin with 60.0 % [64]. The resistance rate to ciprofloxacin was 66.7 % which is in agreement with a previous study showing high resistance [51, 64], but in contrast to another study with only 9.5 % [69]. Resistance to nalidixic acid was 88.9 % which is similar to several aforementioned reports [51, 64, 68, 70]. However, other studies found either low resistance [71, 72] or none at all [65]. Resistances to tetracycline was higher (77.8 %) than previously reported (32.0 %) [72], but it was lower than in isolates recovered from conventionally grown turkeys [68].

The gene loci responsible for antibiotic resistance were detected in all resistant isolates to ciprofloxacin and erythromycin and 66.7 % of resistant isolates to tetracycline. Ciprofloxacin resistance among *C. jejuni* and *C. coli* isolates was conferred by threonine-to-isoleucine mutation of amino acid 86 of the *gyr*A protein (Thr-86-Ile), a finding that is in agreement with other previous studies [73–76]. Tetracycline resistance was attributed to the presence of the *tet*(O) gene [51]. All resistant isolates in this study were carrying *tet*(O); none of the susceptible isolates gave a positive result using specific PCR.

## Conclusions

To the best of our knowledge we present here the first detailed characterization of Vietnamese *Campylobacter* isolates regarding genetic diversity, virulence-associated genes and antibiotic susceptibility. The limitation of our study is the small number of isolates. Further studies are needed to improve our knowledge about the epidemiology and relevance of *Campylobacter* for human health in Vietnam.
